# Correction: Papukashvili et al. “Attenuation of Weight Gain and Prevention of Associated Pathologies by Inhibiting SSAO” *Nutrients*, 2020, *12*, 184

**DOI:** 10.3390/nu12071968

**Published:** 2020-07-02

**Authors:** Dimitri Papukashvili, Nino Rcheulishvili, Yulin Deng

**Affiliations:** 1 School of Life Science, Beijing Institute of Technology, Beijing 100081, China; dimitri@bit.edu.cn (D.P.); nino@bit.edu.cn (N.R.); 2 Beijing Key Laboratory for Separation and Analysis in Biomedicine and Pharmaceuticals, Beijing 100081, China

The authors wish to make a correction to the published paper [1] in Section 1 to make the sentence more understandable. The edited sentence is the following: “It is noteworthy that an enzyme semicarbazide-sensitive amine oxidase (SSAO), also known as vascular adhesion protein-1 (VAP-1), which is responsible for deamination of the primary amines such as methylamine and converts them into cytotoxic aldehydes (e.g., formaldehyde), ammonia, and hydrogen peroxide, is found to be associated with obesity and related diseases.”

In Section 3, Table 1, the edition is needed. In Caffeine row (IC_50_), misspelled “nM” is corrected into “mM”. The edited version of the Table 1 is given below:

In Section 4, an amendment is needed to solve the inconsistency in the sentence. The corrected version of the sentence is the following: “Olivieri and Tipton have revealed the inhibitory concentration (IC) of caffeine intake—0.1–10 mM (IC_50_ = 0.8 ± 0.3 mM). As stated in the studies, the recommended daily dose of caffeine (400 mg) for adults is consistent with 1-4 cups of regular coffee and is not associated with unfavorable effects on health.”

Here is the specific response of the authors to the concerns that were raised in the comment [2]. Although Olivieri et al. have demonstrated that caffeine exerts the inhibition of bovine serum SSAO with a concentration of IC_50_ of 0.8 ± 0.3 mM (in particular, in the concentration range of 0.1–10 mM inhibits SSAO activity) [3] that exceeds the concentration range of caffeine in serum of humans, an animal study conducted by Che et al. revealed that caffeine might be a promising inhibitor of SSAO even with low (nonlethal) concentrations [4]. However, no human studies have been carried out in terms of using caffeine as an inhibitor of enzyme SSAO that may show the different range of caffeine’s inhibitory concentration.

Although Westerterp-Plantenga manifested weight loss and weight maintenance when caffeine together with green tea extract was administered [5], in our article, we present an evidence-based discussion of the weight-gain diminishing capacity of caffeine [6,7,8,9,10]. In addition, the current manuscript does not focus on the state of the body (whether the body is in resting or active state) during caffeine intake and its action. The main point of our article is to provide data about weight gain attenuation by inhibiting SSAO, and it discusses the convenience of some potential inhibitor substances. Moreover, caffeine is less risky and the only popular natural compound with the potential capacity to inhibit the enzyme SSAO. While caffeine is already famous with its antilipolytic effects [6,7,8,9,10], evincing another possible beneficial property [3,4] makes sense. Thus, the assumption of the dual beneficial role of caffeine in this article remains rational. Consequently, we believe human studies need to be carried out on SSAO inhibition by caffeine. The authors appreciate the opportunity to respond to the comment by Willson [2] and hope the information given is satisfactory and clear.

The authors apologize to the readers for any inconvenience caused by this amendment. This amendment does not affect the results or conclusion of the manuscript in any way. The original manuscript will remain online on the article webpage with a reference to this correction.

## Figures and Tables

**Table 1:** Description of semicarbazide-sensitive amine oxidase/vascular adhesion protein-1 (SSAO/VAP-1) inhibitors according to chemical and pharmacological properties.

Names of VAP-1/SSAO Inhibitors	Chemical Structure/Formula	Molecular Weight/Molar Weight	Solubility	Pharmacokinetic Profile		IC_50_	Efficacy/Anti-Obesity Property	Toxicity	Source
				Oral Dose (Rat/Mouse)	i.v./i.p. Dose (Rat/Mouse)				
PXS-4728A/BI1467335	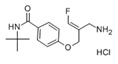 C_15_H_22_ClFN_2_O_2_	316.8014 kDa	DMSO and H_2_O >10 mg/mL	6 mg·kg^−1^ 10 mg·kg^−1^	3 mg·kg^−1^ 5 mg·kg^−1^	5 nM	Potent and orally available inhibitor of VAP-1, showing >500-fold selectivity for VAP-1/SSAO over all the related human amine oxidases. Diminishes lung inflammation. It is in clinical trials for the treatment of cardio-metabolic diseases. It shows significant reduction of body weight gain in rabbits. Axon Medchem, Groningen, Netherlands	No/Low	Wang et al. Schilter et al. Kim et al.
PXS-4681A	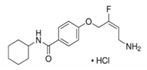 C_17_H_24_ClFN_2_O_2_	342.84 kDa	H_2_O 2 mg/mL	20 mg·kg^−1^ 2 mg·kg^−1^	10 mg·kg^−1^ 2 mg·kg^−1^	<10 nM	Potent and highly selective irreversible inhibitor of SSAO/VAP-1 that exhibits anti-inflammatory effects in vivo. It is a derivative of Mofegiline. PXS-4681A was used to inhibit LPS induced brain inflammation. Sigma-Aldrich, St. Louis, USA	No/Low	Becchi et al. Foot et al.
Semicarbazide	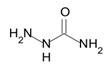 CH_5_N_3_O	75.07 g/mol	N/A	N/A	N/A	N/A	An irreversible and probably suicide SSAO inhibitor. It limits weight gain and fat accumulation. Sigma-Aldrich, Saint Quentin Fallavier, France	Yes/High	Mercader et al.
LJP-1586	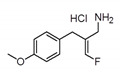 C_11_H_15_ClFNO	231.69 kDa	DMSO	10 mg/kg	N/A	4–43 nM	Potent, selective, and orally active inhibitor of SSAO activity, inhibiting vascular adhesion protein 1 (VAP-1) activity and decreasing the density of macrophages in inflamed atherosclerotic plaques in mice LJP. Glixx Laboratories Inc., Hopkinton, USA	Yes	O’Rourke et al.
Caffeine	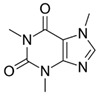 C_8_H_10_N_4_O_2_	194.19 g/mol	H_2_O	N/A	N/A	0.8 ± 0.3 mM	Efficiency of caffeine on adipose and aorta is especially high. It can play an important role in treating diseases associated with SSAO activities. Independently of SSAO inhibition, it is found to be effective in losing weight.National Institute for Drug Control, Beijing, China	No/Low	Che et al. Zheng et al.
Simvastatin	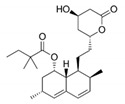 C_25_H_38_O_5_	418.6 g/mol	DMSO and H_2_O	N/A	20 mg·kg^−1^	N/A	Simvastatin blocks SSAO/VAP-1 release, among other known actions, therefore preventing this cascade of events.Sigma-Aldrich, Madrid, Spain	Yes	Sun et al.
Phenylhydrazine	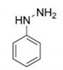 C_6_H_8_N_2_	108.14 g/mol	H_2_O	N/A	N/A	30 nM	Irreversible SSAO inhibitor.Shows diminishing body weight gain.Sigma-Aldrich, Poole, UK	Yes/High	Carpene et al. Lizcano et al.
Phenelzine	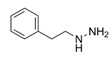 C_8_H_12_N_2_	136.19 g/mol	H_2_O	30 mg·kg^−1^	88.9 µmol/kg	N/A	Potent inhibitor of SSAO.Shows attenuation of adiposity.Sigma-Aldrich, Saint Quentin Fallavier, France	Yes	Carpene et al.
